# The role of phosphorylation in calmodulin-mediated gating of human AQP0

**DOI:** 10.1042/BCJ20230158

**Published:** 2024-01-04

**Authors:** Stefan Kreida, Jennifer Virginia Roche, Julie Winkel Missel, Tamim Al-Jubair, Carl Johan Hagströmer, Veronika Wittenbecher, Sara Linse, Pontus Gourdon, Susanna Törnroth-Horsefield

**Affiliations:** 1 Department of Biochemistry and Structural Biology, Lund University, Lund, Sweden; 2 Department of Biomedical Sciences, Copenhagen University, Copenhagen, Denmark; 3 Department of Experimental Medical Science, Lund University, Lund, Sweden

**Keywords:** aquaporin, gating, membrane protein calmodulin, phosphorylation, protein–protein interaction, water permeability

## Abstract

Aquaporin-0 (AQP0) is the main water channel in the mammalian lens and is involved in accommodation and maintaining lens transparency. AQP0 binds the Ca^2+^-sensing protein calmodulin (CaM) and this interaction is believed to gate its water permeability by closing the water-conducting pore. Here, we express recombinant and functional human AQP0 in *Pichia pastoris* and investigate how phosphorylation affects the interaction with CaM *in vitro* as well as the CaM-dependent water permeability of AQP0 in proteoliposomes. Using microscale thermophoresis and surface plasmon resonance technology we show that the introduction of the single phospho-mimicking mutations S229D and S235D in AQP0 reduces CaM binding. In contrast, CaM interacts with S231D with similar affinity as wild type, but in a different manner. Permeability studies of wild-type AQP0 showed that the water conductance was significantly reduced by CaM in a Ca^2+^-dependent manner, whereas AQP0 S229D, S231D and S235D were all locked in an open state, insensitive to CaM. We propose a model in which phosphorylation of AQP0 control CaM-mediated gating in two different ways (1) phosphorylation of S229 or S235 abolishes binding (the pore remains open) and (2) phosphorylation of S231 results in CaM binding without causing pore closure, the functional role of which remains to be elucidated. Our results suggest that site-dependent phosphorylation of AQP0 dynamically controls its CaM-mediated gating. Since the level of phosphorylation increases towards the lens inner cortex, AQP0 may become insensitive to CaM-dependent gating along this axis.

## Introduction

Light reaches the retina of the eye, allowing us to perceive the world. Before it does, it passes through the lens, which focuses the light, and makes the image sharp. A lens has many unique properties, which are necessary for its function. It is transparent, has an elevated and even refractive index and can change its shape to focus incoming light, a process known as accommodation. Anatomically, the lens is made up of concentrically arranged, tightly packed lens fibre cells of different degrees of maturation. New cells are formed at the lens epithelia with the oldest, most mature cells gradually being pushed into the middle of the lens [1,2]. Fibre cells are hexagonal and elongated and as they mature lose their organelles and nuclei and become increasingly filled with crystalline proteins [3–5].

The membrane intrinsic water channel aquaporin-0 (AQP0) plays an important role in the function of the lens. It belongs to the family of orthodox water channels that selectively allow water to pass through cellular membranes along an osmotic gradient [6]. It is the only aquaporin in lens fibre cells [7] where it constitutes >50% of all membrane proteins [8,9]. Mutations that impair AQP0 function are known to cause cataract and affect lens structure [10–17]. AQP0 is believed to have a dual role in the lens, acting as a water channel as well as mediating cell-to-cell contacts in junctions [18–20]. In consort with ion pumps, AQP0 directs water flow, allowing for nutrient influx into the lens and lens accommodation (caused by water in- or efflux) [1,21,22]. As an adhesion molecule, AQP0 is spread across the plasma membrane of fibre cells [23,24] and interactions between C-terminally truncated AQP0 molecules hold neighbouring membranes tightly together, keeping the distance between membranes below the wavelength of ambient light [2,25–28]. The propensity of truncated AQP0 to self-organize into 2D crystals *in vitro* [29] suggests that a part of the AQP0 population in the core could be in a crystalline state, thereby contributing in itself to the refractive power of the lens.

**Figure 1.**
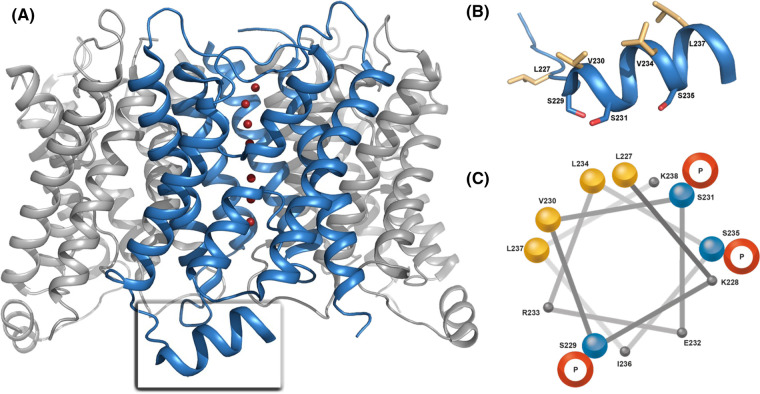
AQP0 structure. (**A**) Structure of bovine AQP0 (PDB code 1YMG [31]) showing the tetramer viewed from the side of the membrane. Each monomer (blue) holds a water channel at its centre, formed by six transmembrane helices and a seventh pseudo-helix. Water molecules are represented as red spheres. The C-terminus extends into the cytosol and forms a short C-terminal helix, which holds the CaM-binding site (**B**) zoom-in and (**C**) helical wheel projection of the C-terminal helix showing its amphipathic character. The hydrophobic face formed by L227, V230 (I230 in human AQP0), L234 and L237 is crucial for the interaction with CaM, whereas the S229, S231 and S235 are located on the other side. Helical wheel projection was generated from rzlab.ucr.edu and the aesthetics of this was modified.

Aquaporins form tetramers and each monomer has at its centre a water pore, formed by six transmembrane helices and a seventh pseudo-transmembrane helix, comprised of two shorter helices in loops B and E that dip into the membrane from either side (Figure 1A). All human orthodox aquaporins have two copies of an asparagine–proline–alanine (NPA)-motif in the centre of the pore and a selectivity filter (first constriction site) at the pore's extracellular entrance, which together provide selectivity for water [30]. In addition, the AQP0 pore has a second constriction site (CSII) located towards the intracellular side, which is involved in the gating of the channel [26,31,32]. The position of CSII along the water-conducting pore coincides with that of other gated AQPs [33–35], and thus seems to be a conserved structural feature in AQP regulation by gating. Gating of AQP0 has been proposed to be triggered by pH [36,37], junction-formation [26,38], and binding of calmodulin (CaM) [32,37,39–41]. CaM is a ubiquitous Ca^2+^-sensing protein that is known to bind and modulate the activity of numerous interaction partners [42] and is present in the lens [43,44]. Functional studies in *Xenopus* oocytes have shown that CaM and Ca^2+^ decrease the osmotic permeability (*P_f_*) of AQP0 [37,39,41], although it remains to be conclusively shown that this is a result of a direct interaction between the two and not mediated by other CaM-dependent pathways.

The AQP0 C-terminus (residues 222–263) extends into the cytosol and plays an important role in AQP0 regulation; harbouring several post-translational modification sites as well as the proposed CaM-binding site (Figure 1B,C). The latter is located on a short helix formed by residues 223–242 (hereafter referred to as the C-terminal helix), a structural feature that is also present in other mammalian AQPs [30]. The Ca^2+^-dependent interaction between AQP0 and CaM has been established by several studies and it has been suggested that one CaM binds two copies of the AQP0 C-terminal helix, i.e. two CaM binding sites per AQP0 tetramer [32,40,45–47]. We recently used full-length AQP0, recombinantly expressed in *Pichia pastoris*, to quantify this interaction *in vitro* using microscale thermophoresis (MST). Our data showed a strong positive cooperativity, i.e. that CaM binding to the first binding site of AQP0 increases its affinity for the second one, which was not evident when using AQP0 C-terminal peptides [48]. Moreover, structural, computational and functional studies have suggested that CaM may also interact with cytosol-facing loops of the AQP0 tetramer in addition to the C-terminal helix [32,49,50].

Structural studies of bovine and ovine AQP0 [27,31] have shown that the AQP0 C-terminal helix has a very strong amphipathic character (Figure 1B,C) with its hydrophobic side, formed by residues L227, V230, L234 and L237 (bovine numbering), being crucial for the interaction with the hydrophobic binding pocket of CaM [40]. The polar face of the helix, located on the opposite side, contains three phosphorylation sites at S229, S231 and S235, the latter being the most prominent site *in vivo* [36,51–54]. Quantitative studies of the effect of phosphorylation on the interaction between AQP0–C-terminal peptides and CaM have shown that the affinity is reduced [40,46,47,49], which could indicate that phosphorylation sterically blocks CaM from binding. This was further supported by oocyte experiments using AQP0 phospho-mimicking mutants, where phosphorylation was demonstrated to result in AQP0 being locked in either an open (S229D and S231D) or closed (S235D) state with reduced Ca^2+^-sensitivity [49,55]. Moreover, mutations of two proximal glycation sites (K228N and K238N) were shown to reduce the affinity of CaM to AQP0 in insect cell membranes [56], and the same residues were found to cross-link with CaM using a zero-length cross-linker [46]. Molecular dynamics simulations have suggested that AQP0 phosphorylation may not alter the affinity to CaM but instead changes the interaction with an allosteric cytoplasmic loop, thereby mediating pore closure [50]. Taken together, these results indicate that the polar face of the AQP0 C-terminal helix may also be involved in the CaM interaction and that phosphorylation (or glycation) disturbs CaM binding.

In this work, we use functional studies as well as protein–protein interaction studies to investigate how phosphorylation affects CaM-mediated regulation of AQP0. Using full-length AQP0 and AQP0 phospho-mimicking mutants (S229D, S231D and S235D) reconstituted in proteoliposomes, we show that CaM directly inhibits AQP0 and that phosphorylation at any site abolishes this inhibition. Interaction studies using MST and surface plasmon resonance (SPR) technology further show that, while AQP0 S229D and S235D are unable to bind CaM or have significantly reduced affinity, AQP0 S231D interacts with similar affinity as wild type, but in a different mode, leaving the pore open. Based on these results we suggest a regulatory model whereby the CaM-mediated gating of AQP0 is dynamically regulated by AQP0 phosphorylation in a site-dependent manner.

## Results

### AQP0 is not phosphorylated or truncated in yeast

**Figure 2.**
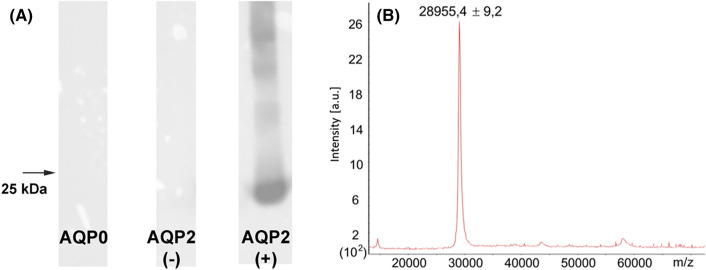
Phosphorylation and truncation analysis of recombinantly expressed human AQP0. (**A**) Phos-blot using using Phos-tag BTL-111 conjugated to HRP, showing that AQP0 is not phosphorylated in *P. pastoris*. AQP2 expressed in *P. pastoris* was used as positive control and showed a clear band at 25 kDa (monomer) as well as for higher oligomeric states. These disappeared if AQP2 was treated with alkaline phosphatase (negative control). (**B**) Representative mass spectrum of intact AQP0 as measured by linear mode MALDI spectrometry. The spectrum shows one distinguishable peak of 289 554 ± 9.2 [M + H^1+^] (*n* = 3), which is in agreement with the predicted mass of 6× His-tagged AQP0 of 28 944.6 Da.

Human AQP0 containing a C-terminal His-tag was expressed in *P. pastoris* and purified as previously described [48]. In lens fibre cells, AQP0 is subject to post-translational modifications, including phosphorylation and C-terminal truncation. Since *P. pastoris* has been observed to phosphorylate recombinant proteins, we tested the phosphorylation state of our recombinant AQP0 using Phos-blot (Figure 2A), using human AQP2, which is known to be phosphorylated when expressed in *P. pastoris* as a positive control [57]. As expected, clear bands could be observed for AQP2 at the appropriate size, indicating phosphorylation. These bands were removed if the sample was treated with bovine alkaline phosphatase. In contrast, no bands could be seen when loading an equal amount of AQP0, showing that, when expressed in *P. pastoris*, AQP0 is not phosphorylated.

To investigate whether AQP0 is C-terminally truncated in yeast, thereby possibly removing the CaM-binding site, we analysed the intact protein using linear mode MALDI mass spectrometry (Figure 2B). This resulted in a spectrum with one distinguishable peak of 28 955.4 ± 9.2 Da [M + H^1+^] (*n* = 3). This corresponds well with the predicted mass of the His-tagged protein is 28 944.6 Da, confirming that AQP0 retains its C-terminus in *P. pastoris*.

### CaM reduces the water permeability of AQP0 in proteoliposomes

**Figure 3.**
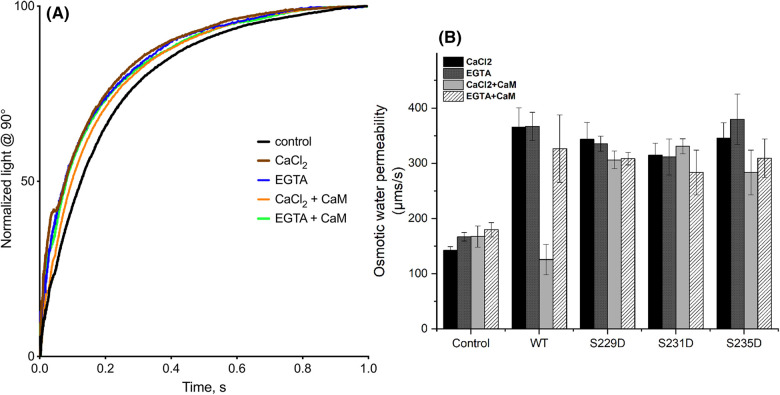
CaM-dependency of AQP0 water permeability and the effect of phosphorylation. (**A**) Typical data and curve fit from the proteoliposome assay for wild-type AQP0. Each curve is the averaged trace from one reconstitution. The data could be described by a double exponential function, from which the osmotic water permeability (*P_f_*) was calculated. All experiments were done in triplicates, using a LPR of 10. (**B**) *P_f_* for wild-type AQP0 and AQP0 phospho-mimicking mutants reconstituted into proteoliposomes in the presence of CaCl_2_, EGTA, CaCl_2 _+ CaM and EGTA + CaM, respectively. The *P_f_*-values for AQP0-containing proteliposomes are adjusted for reconstitution efficiency (Supplementary Figure S1). CaM significantly reduces the permeability of wild-type AQP0 in a Ca^2+^-dependent manner (*P* < 0.001), an effect that is reversed in the presence of EGTA. AQP0 S229D, S231D and S235D have similar water permeability as wild-type AQP0 but are insensitive to CaCl_2 _+ CaM. All AQP0 constructs show significantly higher water permeability than control empty liposomes (*P* < 0.001) demonstrating that they are all functional water channels.

Previous studies in *Xenopus* oocytes have suggested that CaM reduces the water permeability of AQP0 in a Ca^2+^-dependent manner [37,39]. In these studies, the involvement of CaM in AQP0 gating was concluded from the effect of CaM inhibitors and the co-expression of a ‘crippled’ CaM mutant. However, oocytes are complex cellular systems, and it cannot be excluded that AQP0 gating is controlled by a CaM-dependent pathway rather than by direct interaction with CaM. To verify that AQP0 is indeed directly regulated by CaM, we performed water permeability assays of AQP0 reconstituted in proteoliposomes. The proteoliposomes were generated using a lipid-to-protein ratio (LPR) of 10 in the presence of Ca^2+^, EGTA, Ca^2+^/CaM and EGTA/CaM, respectively. The comparably low LPR was selected due to the poor water permeability of AQP0 [58,59]. Proteoliposomes were rapidly mixed with a hyperosmotic solution and the increased light (due to shrinking of the liposomes) was measured at a 90° angle on a SX-20 stopped-flow spectrometer for a duration of 5 s (Figure 3A). The rate constants were calculated from fitting a two-exponential function to a normalised average of three (see experimental section) and used to calculate the osmotic water permeability (*P_f_*). The *P_f_*-values were adjusted for differences in protein amounts by quantifying the amount of proteins in the liposomes using Western blots (Supplementary Figure S1).

As seen in Figure 3 and Supplementary Table S1, liposomes containing AQP0 had a significantly higher water permeability than control empty liposomes, the *P_f_*-values were 365 ± 35.0 and 142.2 ± 6.79 µm s^−1^, respectively, proving that the here purified AQP0 is a functional water channel (*P* < 0.001). In the presence of Ca^2+^/CaM, AQP0 water permeability was reduced to similar levels as control liposomes (*P_f_* was 126 ± 27.7 µm s^−1^) an effect that was abolished when EGTA was added instead of Ca^2+^. In contrast, Ca^2+^ or EGTA without CaM had no effect. Taken together, this shows that CaM directly controls AQP0 water permeability in a Ca^2+^-dependent manner.

### AQP0 phosphorylation abolishes CaM-dependent inhibition of water permeability

To investigate the effect of phosphorylation on AQP0 water permeability and its regulation by CaM, we used mutagenesis PCR to introduce the phospho-mimicking mutations S229D, S231D and S235D in our human AQP0 construct. These mutants were expressed, purified and assayed for CaM-dependent water permeability using the same protocols as for wild-type AQP0. All mutants displayed similar water-conducting ability as wild-type AQP0, indicating that phosphorylation *per se* does not affect the AQP0 pore (Supplementary Figure S2 and Table S1, Figure 3B). However, in contrast to wild-type AQP0, neither of the mutants displayed altered water permeability in the presence of Ca^2+^/CaM. This suggests that AQP0 phosphorylation at either site abolishes CaM-dependent inhibition of water permeability, thus providing an additional level of control for the CaM-mediated gating of AQP0 in the lens.

### Phosphorylation of AQP0 controls CaM binding

To study if phosphorylation abolishes inhibition by preventing CaM binding, we quantified the interaction between AQP0 phospho-mimicking mutants and CaM using MST. We have previously used this method to show that one CaM molecule binds two copies of AQP0 in a highly cooperative manner [48]. Here we employ the same experimental setup whereby CaM carrying an S17C mutation was used, allowing it to be labelled with the cysteine-reactive dye Alexa^488^. For the experiment, dilution series of wild-type AQP0 and AQP0 mutants were created and mixed with a constant concentration of CaM-Alexa^488^, after which MST traces were recorded (Supplementary Figure S3).

**Figure 4.**
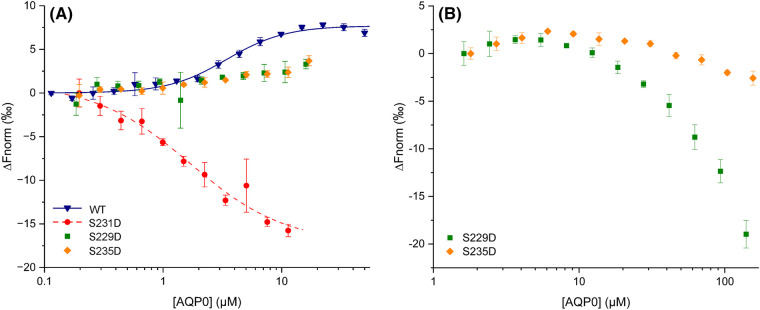
MST analysis of the effect of phosphorylation on the interaction between AQP0 and CaM. (**A**) MST data for wild-type AQP0 (previously published in [48]), AQP0 S229D, AQP0 S231D and AQP0 S235D. For AQP0 S229D and S235D, binding was not observed. In contrast, a binding curve was obtained for AQP0 S231D which could be described by a one-to-one binding equation with an estimated *K_D_* of 1.49± 0.48 µM. This affinity is similar to wild-type AQP; however, in this case, the data were best described by a two-to-one model (*K_D_*_1_ = 40 µM, *K_D_*_2_ = 2.5 µM) with strong cooperativity between the sites. Moreover, the two binding curves are in opposite directions, indicating biophysical differences between the complexes. (**B**) MST data for AQP0 S229D and S235D show that there is no interaction at AQP0 concentrations below 50 µM. At very high AQP0 concentrations, a decrease in MST signal can be observed; however, this does not reach a plateau within the range of AQP0 concentrations used here (up to 150 µM) and is, therefore, interpreted as non-specific or low affinity binding without physiological significance.

As seen in Figure 4A, binding curves were obtained for wild-type AQP0 and AQP0 S231D. In contrast, AQP0 S229D and S235D did not show any binding within the same AQP0 concentration range. At very high AQP0 concentrations (>50 µM), the MST signal decreases for both these mutants (Figure 4B); however, since this does not reach a plateau within our experimental range (up to 150 µM), we interpret this as non-specific binding or very low affinity binding without physiological significance.

Interestingly, the MST results for wild-type AQP0 and AQP0 S231D suggest that although they bind with similar affinity, there are differences in the binding mode. Specifically, the data for wild-type AQP0 were best described by one CaM binding two AQP0 molecules with *K_d_*_1_ of 40 µM and *K_d_*_2_ of 2.5 µM, with strong cooperativity between the sites [48]. In contrast, the binding curve for AQP0 S231D could be fitted to a one-to-one binding model with a *K_d_*-value of 1.49 ± 0.48 μM. Moreover, the AQP0 S231D MST signal decreased, rather than increased with incrementing AQP0 concentration. Since thermophoresis depends on the size, charge, solvation entropy and conformation [60], this suggests that there are biophysical differences in the complexes that are formed. Our MST results, therefore, suggest that AQP0 S231D binds CaM with similar affinity as the wild-type AQP0 site with higher affinity, but with a different binding mode.

**Figure 5.**
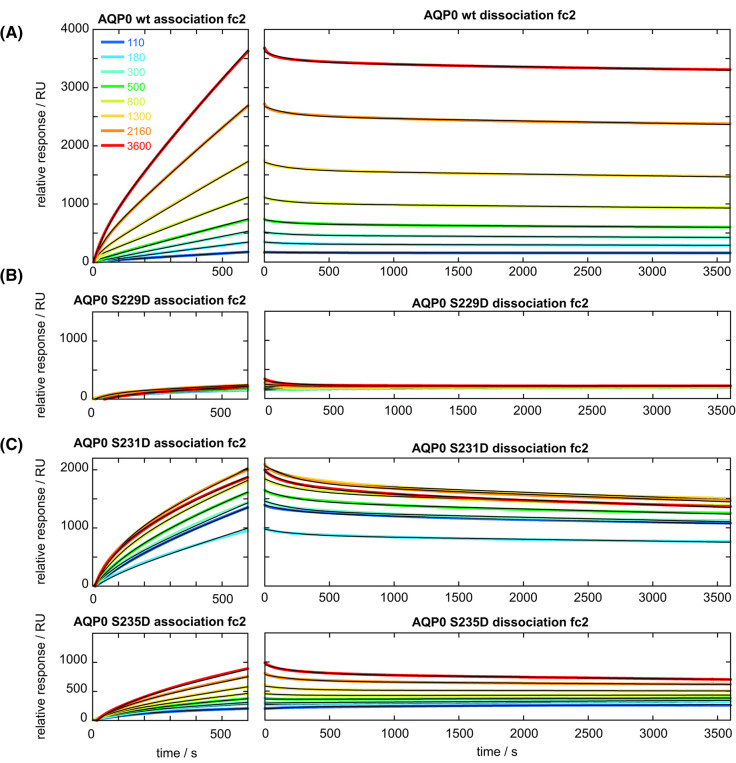
SPR analysis of the effect of phosphorylation on the interaction between AQP0 and CaM. SPR data for (**A**) wild-type AQP0, (**B**) AQP0 S229D, (**C**) AQP0 S231D and (**D**) AQP0 S235D). The data are from flow cell 2 after blank subtraction and shown with equal *y*-scale with the colour codes for the concentration in nM given in the upper left panel. The left panels show association phase data under the injection of AQP0 in constant flow, and the right panels show the dissociation phase data under the following buffer flow. The black lines are fitted bi-exponential curves (see Materials and methods). Wild-type AQP0 and AQP0 S231D bind CaM with similar affinity (√(*K_D_*_1_ *K_D_*_2_) is 550 ± 30 nM for wt and 590 ± 40 nM for S231D), whereas the affinity for AQP0 S235D was significantly lower (5.0 ± 0.4 µM, *P* < 0.001) and no binding was observed for AQP0 S229D.

The interaction between the different AQP0 constructs and CaM was further explored using SPR. For this purpose, a series of AQP0 solutions were injected over a sensor chip with one blank channel and three channels with immobilised CaM, thus providing three data sets. The data from one channel with fitted curves are shown in Figure 5 and the other two in Supplementary Figure S4. The fitted parameters are shown in Table 1. For wild-type AQP0, AQP0 S231D and AQP0 S235D binding was observed, whereas an interaction between CaM and AQP0 S229D could not be detected by SPR. While the data were best described by a binding model with two processes (see Materials and Methods), the first process (described by *K_D_*_1_, *k*_off1_ and *k*_on1_) represented only 5–10% of the total amplitude for all three constructs, and the main process is described by the values of *K_D_*_2_, *k*_off2_ and *k*_on2_. It is not possible to tell whether the two processes are associated with one or two AQP0 protomers interacting with the same CaM on the chip, as expected for a cooperative process, or one AQP0 protomer interacting with one or two CaM molecules on the chip (an avidity effect). The geometric means of *K_D_*_1_ and *K_D_*_2_, √(*K_D_*_1_ *K_D_*_2_), were, therefore, used for comparison of the overall CaM affinity. For wild-type AQP0 and AQP0 S231D, √(*K_D_*_1_ *K_D_*_2_) was calculated to be 550 ± 30 and 590 ± 40 nM, respectively, in good agreement with the similar affinity observed for these two constructs in the MST experiment. In contrast, the overall affinity for AQP0 S235D was significantly lower (√(*K_D_*_1_ *K_D_*_2_) = 5.0 ± 0.4 µM) (*P* < 0.001). When the affinity constants of only the major process, *K_D_*_2_, are compared we find a slight reduction in affinity for AQP0 S231D (*K_D_*_2_ = 700 ± 30 nM) compared with wild-type AQP0 (*K_D_*_2_ = 150 ± 5.0 nM) (*P* < 0.001) and substantially lower affinity for AQP0 S235D (*K_D_*_2_ = 10 ± 2.0 µM) (*P* < 0.001). Although the origin of the two processes is not clear, we note that the difference in affinity is well above the limit for positive cooperativity (*K_D_*_2_ < 4 *K_D_*_1_) for wild-type AQP0, and above the limit for S231D [61], suggesting a higher degree of cooperativity for wild-type AQP0.

**Table 1.** Fitted rate constants and calculated equilibrium dissociation constants for the AQP0–CaM interaction observed by SPR

AQP0 variant	*k*_off1_ (s^−1^)	*k*_on1_ (M^−1^s^−1^)	*K_D_*_1_ (µM)	*k*_off2_ (s^−1^)	*k*_on2_ (M^−1 ^s^−1^)	*K_D_*_2_ (µM)	√(*K_D_*_1_ *K_D_*_2_) (µM)
WT	0.007 ± 0.002	3200 ± 1000	2.2 ± 0.8	2.0 ± 0.5 × 10^−5^	130 ± 30	0.15 ± 0.05	0.55 ± 0.03
S229D	—	—	—	—	—	—	—
S231D	0.004 ± 0.001	8000 ± 2000	0.5 ± 0.2	4.5 ± 1.0 × 10^−5^	64 ± 20	0.7 ± 0.3	0.59 ± 0.04
S235D	0.004 ± 0.001	1300 ± 300	3.0 ± 1.0	3.0 ± 0.5 × 10^−5^	3.0 ± 0.5	10 ± 2	5.0 ± 0.4

The minor and major processes are denoted 1 and 2, respectively. The error is the standard deviation.

Taken together, these binding studies show that AQP0 S231D binds CaM with similar affinity as wild-type AQP0 but with a different binding mode, whereas AQP0 S229D and AQP0 S235D bind with significantly lower affinity or not at all. Since the permeability assay showed that the water conductance through AQP0 S231D is insensitive to CaM (Figure 3B), we propose that the mode in which CaM interacts with this mutant does not result in pore gating.

## Discussion

Many studies have explored how the AQP0–CaM interaction relates to the AQP0 function. In AQP0-expressing oocytes as well as native AQP0 incorporated into proteoliposomes, Ca^2+^ decreased the osmotic permeability (*P_f_*) twofold and this Ca^2+^ dependency was removed in the presence of CaM inhibitors [37,39,41]. Together with NMR stoichiometry studies on full-length protein, binding studies using peptides [40] and full-length AQP0 [48] as well as an electron microscopy structural model of the AQP0–CaM complex [32], this has resulted in a model whereby each AQP0 tetramer binds two CaM molecules, which block the water pores. The AQP0 proteoliposome permeability assay presented here supports this model and we also observe a twofold decrease in permeability in the presence of both CaM and Ca^2+^. In our experimental setup, we use recombinant AQP0 that is incorporated into proteoliposomes and we ensure that AQP0 is not subject to phosphorylation or C-terminal truncation during expression in yeast. By providing these controls and by eliminating cellular components other than CaM for the functional assay this result is an important validation of the model above. It should be noted, however, that permeability studies on lens fibre cells show that Ca^2+^ increases water permeability rather than decreasing it [36]; thus illustrating the complexity of cellular systems for functional assays.

Previous studies of how phosphorylation affects the interaction between AQP0 and CaM have primarily been done using peptides corresponding to the CaM-binding site (AQP0 C-terminal helix). These have shown that phosphorylation at S229, S231 and S235, respectively, abolishes CaM binding [40,46]. We recently showed that there is a difference in binding between AQP0 peptides and full-length AQP0, where cooperativity could only be observed when full-length protein was used [48]. In light of this, we, therefore, set out to explore how phosphorylation affects CaM binding to full-length AQP0, using MST and SPR. In agreement with reported peptide data [46], we show that both the S229D- and S235D-mutations abolish CaM binding in MST. In SPR, an interaction between AQP0 S229D and CaM could not be detected while AQP0 S235D bound CaM with significantly lower affinity than wild-type AQP0. This discrepancy in CaM binding for AQP0 S235D is likely due to methodological differences, for example, the surface-immobilisation of CaM in SPR. For AQP0 S229D, the lack of CaM binding has been shown previously in the full-length context, with oocytes expressing AQP0 S229D in their membrane binding less fluorescently labelled CaM than those expressing wild-type AQP0 [55]. However, since differences in membrane expression levels between wild-type AQP0 and AQP0 S229D were not taken into account, it is difficult to assess if this truly reflected a difference in CaM affinity.

In contrast with the peptide binding studies, our results show that full-length AQP0 S231D binds CaM but in a different manner than wild-type AQP0. The disagreement between peptide and full-length AQP0 binding studies for AQP0 S231D as well as wild-type AQP0 gives further support to the notion that regions outside the AQP0 C-terminal helix are involved in CaM binding. Based on the 25 Å electron microscopy model of the AQP0–CaM complex it was suggested that the interaction is partially driven by electrostatics between the negatively charged CaM and the positively charged cytosolic face of AQP0 [32], interactions that would be absent from binding data collected using AQP0 C-terminal helix peptide fragments. Molecular and Brownian dynamics simulations based on this structural model and comparison of wild-type AQP0 with phosphorylated AQP0 showed that CaM binds allosterically to residues 150–156 of loop D, which is rich in arginines [50]. Based on this, it was proposed that phosphorylation of S229 and S235 would not affect the affinity, but rather the binding mode between loop D and CaM. Our data support the hypothesis that phosphorylation of AQP0 breaks this electrostatic interaction but instead identifies S231 as the residue likely to be responsible for this effect.

In addition to interaction studies, the effect of phosphorylation and CaM on AQP0 function has been investigated using phospho-mimicking AQP0 mutants expressed in oocytes [55]. In this study, AQP0 S229D and S231D were locked in a high *P_f_*-state that was insensitive to Ca^2+^ and CaM. In contrast, S235D displayed low water permeability which increased at high (5 mM) Ca^2+^ concentrations or in the presence of CaM inhibitors. From these studies, the authors concluded that phosphorylation at S229 and S231 abolishes CaM binding and gating. They further suggested that phosphorylation of S235 mediates pore closure on its own and that CaM may still bind at a secondary site. However, in a complex system such as oocytes, the addition of CaM-effectors and Ca^2+^ are likely to affect many cellular pathways, including membrane translocation wherefore the resulting effect may not be direct. Moreover, natively expressed AQP0 may contain post-translational modifications that could influence CaM binding and inhibition, most notably phosphorylation and C-terminal truncation.

To avoid these complications, we studied the water permeability of recombinant AQP0 phospho-mimicking mutants in proteoliposomes, allowing us to control which proteins are present as well as their post-translational modification status. Our studies show that all three phospho-mimicking mutants have similar water permeability as wild-type AQP0 (Figure 3B) but were unaffected by CaM and Ca^2+^. For AQP0 S229D and S231D, this is in agreement with the oocyte studies. The lack of inhibition of AQP0 S231D supports that CaM binds in a different manner, as suggested by our binding studies, indicating that CaM is unable to close the pore. For AQP0 S235D our result differs from what has been previously described both in terms of the open/closed state of the channel and Ca^2+^/CaM-sensitivity [55]. Since our binding studies showed no or reduced binding of CaM, we propose that the observed low permeability of AQP0 S235D in oocytes is a result of differences in membrane translocation, a process that may involve Ca^2+^ and/or CaM. CaM-dependent pathways have been demonstrated to play a role in the translocation of several other AQPs, including AQP1 [62], AQP2 [63], AQP4 [64,65] and AQP5 [66]. Furthermore, phosphorylation of S235 has been shown to be necessary for correct trafficking between the trans-Golgi network and the plasma membrane [67], suggesting that this site does play an important role in determining AQP0 sub-cellular localisation.

**Figure 6.**
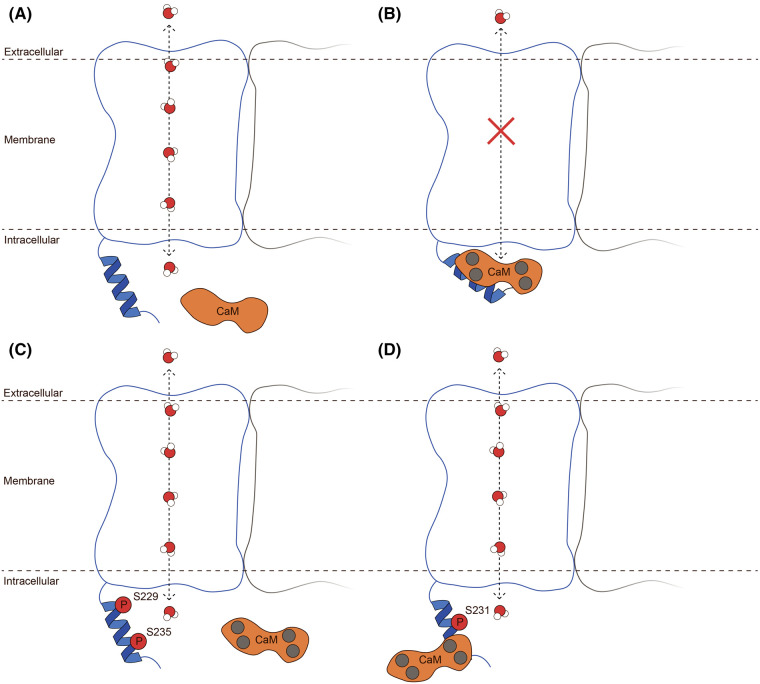
Phosphorylation-dependent gating of AQP0 by CaM. Schematic figure describing the proposed model of how phosphorylation of AQP0 affects gating by Ca^2+^-bound CaM. (**A**) In the absence of Ca^2+^, CaM (orange) is unable to bind and AQP0 is permeable to water. (**B**) Binding of Ca^2+^ (grey spheres) to CaM induces its interaction with the AQP0 C-terminal helix (blue), thereby inhibiting water permeability through AQP0. (**C**) AQP0 phosphorylation at S229 or S235 (red spheres) abolishes the binding of Ca^2+^–CaM and the channel remains open. (**D**) AQP0 phosphorylated at S231 is able to bind Ca^2+^–CaM but the binding mode is altered and the water channel remains open.

In summary, we propose a model (Figure 6) where AQP0 can have three states in relation to its phosphorylation status: (1) wild type, non-phosphorylated — AQP0 can interact with CaM and the interaction causes pore closure. (2) pS231 — AQP0 can interact with CaM but the interaction is altered compared with wild type and does not cause pore closure. (3) pS229 or pS235 — CaM is blocked from interacting with AQP0 and the pore is locked in a Ca^2+^-insensitive open state. From a physiological point of view, this is particularly interesting for S235, the main phosphorylation site of AQP0 *in vivo*. Since this residue becomes increasingly phosphorylated towards the lens core [53], this offers a dynamic mechanism for controlling AQP0 gating that via the action of kinases loosens its CaM/Ca^2+^-inhibition towards the centre of the lens. For S231, which is a minor phosphorylation site, the altered CaM binding mode could indicate some other physiological role during the maturation process of AQP0 or the lens; however, more studies will be needed to elucidate this.

## Materials and methods

### Cloning, expression and purification of AQP0 mutants

AQP0 mutants were generated by introducing point mutations into wild-type hAQP0-6×His in pPICZA [48] using a particularly robust two-step mega-primer scheme [68]. In the first step, AOX'1 forward primer and a reverse primer containing the mutation generated a large DNA fragment containing the mutation. The primers used were:

S229D: TTTGGCACCCTTGAGGACAGACAGTCTCTCAGAAATGTCCTTGAGCCGGGGGAAGAG,
S231D: TTTGGCACCCTTGAGGACAGACAGTCTCTCATCAATACTCTTGAGCCGGGGGAAGAG,
S235D: TTTGGCACCCTTGAGGACATCCAGTCTCTCAGAAATACTCTTGAGCCGGGGGAAGAG

The resulting fragments were then used as ‘mega primers’ for a subsequent PCR step.

Cloning and expression of AQP0 mutants were done in *P. pastoris* as previously described [48]. Briefly, cells were grown in a fermenter (Belach Bioteknik) and expression was induced with MeOH for 120 h. Cells were broken using a bead beater (12 × 30 s with 30 s pause) in 50 mM phosphate buffer pH 7.5, 10% glycerol, 1 mM PMSF. Cells were spun down at 10 000×***g*** (30 min). Membranes were extracted from the supernatant after ultracentrifugation at 100 000×***g*** (1 h), washed in 5 mM Tris–HCl pH 9.5, 4 M urea, 2 mM EDTA, ultracentrifugated (2 h) and resuspended in 20 mM Tris–HCl pH 8, 20 mM NaCl, 10% glycerol.

Membranes (0.5 mg ml^−1^) were diluted 1:1 by drop-wise addition of 20 mM Tris pH 8, 300 mM NaCl, 10% glycerol, 2% DDM (Anatrace), complete EDTA-free protease inhibitor cocktail tablet (Roche) and solubilised while stirring for 2 h at 4°C. Non-solubilised debris was spun down at 100 000×***g*** (30 min) and the supernatant was loaded onto a Histrap column (Cytiva), equilibrated with 20 mM Tris pH 8, 300 mM NaCl, 10% glycerol, 10 mM imidazole, 0.05% DDM. The column was washed with 15 column volumes (CVs) of the same buffer but with 75 mM imidazole, followed by 5 CV of buffer with 100 mM imidazole. Protein was eluted with 300 mM imidazole and the relevant fractions were pooled concentrated using 50 MWCO concentrator (Vivaspin). The concentrated sample was loaded onto a Superdex 200 10/300 GL column (Cytiva) equilibrated with 20 mM Tris pH 7.5, 100 mM NaCl, 5% glycerol and 0.05% DDM. Purity was assessed using SDS–PAGE and protein concentration was determined with measurements at A^280^ and *ε* = 39 420 M^−1^cm^−1^.

### Analysis of AQP0 phosphorylation state

The phosphorylation state of AQP0 was investigated using Phos-tag BTL-111 (Wako Pure Chemicals Ltd.). Human AQP2 that has been showed to be phosphorylated during expression in *P. pastoris* previously was used as control [57]. Forty micrograms of AQP0, AQP2 (positive control) and dephosphorylated AQP2 (negative control) were run on SDS–PAGE. Dephosphorylation of AQP2 was done by treatment with 300 U bovine alkaline phosphatase (Sigma) in 5 mM Tris–HCl, 10 mM NaCl, 1 mM MgCl_2_, 0.1 mM DTT, 0.2% OGNG (Anatrace) and at 30°C (2 h). Proteins were blotted on to a PVDF membrane, blocked with BSA (6 h) and treated with a complex of BTL-111 and streptavidin-conjugated HRP, as described previously [57]. The blot was developed with ECL chemiluminescence reagents (GE Healthcare).

### Linear mode MALDI mass spectrometry

To confirm that AQP0 does not get truncated when expressed in *P. pastoris*, we analysed the protein using mass spectrometry. Purified AQP0 (3 mg ml^−1^ in gel filtration buffer) was diluted 10 times with a solution containing 2% acetonitrile and 0.5% trifluoroacetic acid. The sample was spotted on a ground steel plate together with α-cyano-4-hydroxycinnamic acid used as a matrix. Subsequently, the sample was analysed using the positive linear mode on Autoflex speed TOF/TOF MALDI/MS system (Bruker Daltonik GmbH). The method was calibrated with BSA. Peak picking was performed in Mascot Distiller (Matrix Science) version 2.6.3.0 using the default settings.

### Proteoliposome reconstitution and shrinking assay

*Escherichia coli* lipid extract (Avanti polar lipids) was dissolved in chloroform in a glass vial to a concentration of 25 mg ml^−1^, then dehydrated with N_2_ forming a thin lipid bilayer. A final dehydration was performed in a vacuum desiccator (3 h). The resulting lipid film was rehydrated with reconstitution buffer (20 mM Tris–HCl pH 7.5, 200 mM NaCl, with 1 mM CaCl_2_ or 3 mM EGTA with and without 10 µM CaM) with 10 mM (5)6-carboxyfluorescein (Sigma) to a concentration of 20 mg ml^−1^ lipids. The solution was then sonicated in a sonication bath for 3 × 15 min, with 5 min of rest in between. The lipids were flash-frozen in liquid nitrogen. The lipids were subsequently thawed at room temperature, and passed through a 200 nm polycarbonate filter, using an extruder (Mini-Extruder, Avanti), 11 times. The lipids were then diluted to 4 mg ml^−1^ with reconstitution buffer also containing 25% glycerol and 0.4% Nonyl-β-d-Glucoside (NG), after which Triton X-100 was added to the sample to final concentration of 0.02%. Protein was added to the lipid suspension using a weight-based LPR of 10 and each sample was allowed to dialyse overnight at 4°C against reconstitution buffer without CaM. The samples were centrifuged at 57 000×***g*** (1.5 h), and the resulting pellets were resuspended in reconstitution buffer. The experiments were performed on an SX-20 Stopped-Flow Spectrometer system (Applied Photophysics), where the liposomes were mixed with reaction buffer with 800 mOsm NaCl. Data were collected using light with a wavelength of 495 nm and then detected at a 90° angle throughout the reaction, which was measured for 5 s (Figure 3A and Supplementary Figure S2). All data were collected at 18°C. Empty liposomes were used as a negative control. The data were analysed and plotted in Pro-Data Viewer (Applied Photosystems). Each sample was averaged from three readings, normalised and fitted using the following equation:

$$y=y_{0}+A_{1}e^{-k_{1}(x-x_{0})}+A_{2}e^{-k_{2}(x-x_{0})}$$

where *A*_1_ and *A*_2_ are the respective amplitudes and *k*_1_ and *k*_2_ are the fit *k*-rate constants. The smaller *k*-rate, *k*_2_, is unaffected by changes in the reconstitution efficiency, thus concluding that the larger *k*-rate, *k*_1_, represents the *k*-rate of the incorporated AQP0 or mutants.

This *k*-rate constant may then be used to calculate the osmotic water permeability, *P_f_*, from the following equation:

$$P_{f}(\mathrm{cm}\;s^{-1})=\frac{k_{1}}{\left(\frac{S}{V_{0}}\right)\times V_{W}\times C_{\mathrm{out}}}$$

where (*S*/*V*_0_) is the initial surface area to volume ratio of the liposome, *V_W_* is the partial molar volume of water (18 cm^3^ mol^−1^) and *C*_out_ is the external osmolality.

### Reconstitution efficiency determined by Western blot and adjustment of *P_f_*-values

The amount of protein incorporated into the liposomes was determined by Western blot analysis. On one gel, equal amounts of proteoliposomes from the first reconstitution batch were loaded, to create a comparison platform between the constructs AQP0WT, S229D, S231D and S235D. Afterwards, the triplicate reconstitutions of the different constructs were compared on separate gels. The gels were transferred to a membrane (Hybond PVDF, GE Healthcare). The immunoblot was performed using a conjugated antibody against the 6× His-Tag (6× His mAb-HRP conjugated by Takara® Bio Europe AB, Göteborg, Sweden). The signals obtained were quantified using ImageJ 1,50i. To quantify and adjust the *k*_1_ values, the following equation was used:

$$k_{1\;\mathrm{adjusted}}(s^{-1})=\frac{k_{1\;\mathrm{measured}}(s^{-1})-k_{1\;\mathrm{control}}}{\left(\frac{N_{1}}{N_{2}}\right)}$$

where *k*_1measured_ is the value determined from the double exponential fit, *N*_1_ is the factor determined by comparison of the different constructs and *N*_2_ is the factor determined by comparison of triplicates of a single construct (see Supplementary Figure S1 for quantification factors). *N*_1_ was normalised against WT and *N*_2_ was normalised against the first reconstitution of each separate construct. The adjusted *k*_1_ was then used to calculate adjusted *P_f_*-values as described above.

### Microscale thermophoresis and data analysis

Human CaM carrying an S17C mutation was purified as described [69]. Since hCaM lacks cysteines, the mutation introduces a unique site for labelling on the opposite side of the binding cleft that is not likely to interfere with binding. CaM was labelled with Alexa^488^ in a 2:1 Alexa:CaM ratio in 20 mM phosphate buffer pH 8 at RT (3 h). MST experiments were done on a Monolith NT. 115 (NanoTemper Technologies). AQP0 mutants were diluted in a 2:1 dilution series with 20 mM Tris pH 7.5, 100 mM NaCl, 5% glycerol, 0.05% DDM, 1 mM CaCl_2_ and mixed 1:1 with 64 nM or 800 nM CaM-Alexa^488^ (depending on the degree of labelling), resulting in 12 different samples with AQP0 concentrations ranging between 0.18 and 16 µM (series 1)/1.6 and 140 µM (series 2) for S229D, 0.2 and 17 µM for S231D and 0.2 and 17 µM (series 1)/1.8 and 156 µM (series 2) for S235D. For each AQP0 construct, three individual dilution series were prepared. The samples were transferred to premium-coated capillaries and MST data were obtained using an MST and LED power setting of 40% and 20%, respectively. The data for wild-type AQP0 had been measured previously using the same experimental setup [48]. *F*_norm_ was defined as the difference in fluorescence before and after heating (Supplementary Figure S3).

Raw data treatment was done in MO.Affinity analysis software (NanoTemper Technologies) and fitting was done using Origin (OriginLab). For S231, data could be described by a one-to-one binding model:

$$y=S_{1}+(S_{2}-S_{1})\left(\frac{L_{\mathrm{Free}}}{L_{\mathrm{Free}}+K_{D}}\right)$$

$$L_{\mathrm{Free}}=0.5\;(L_{\mathrm{Tot}}-P_{\mathrm{Tot}}-K_{D})+\;\sqrt{0.25{\left(K_{D}+P_{\mathrm{Tot}}-L_{\mathrm{Tot}}\right)}^{2}+L_{\mathrm{Tot}}K_{D}}$$

where *S*_1_ and *S*_2_ are the signal of the unbound and bound form, respectively, *L*_Free_ is the free monomeric (AQP0), *L*_Tot_ is the total monomeric (AQP0), *P*_Tot_ is the total (CaM-Alexa^488^) and *K_d_* is the dissociation constant. For fitting of the wild-type AQP0 data (see [48]).

### SPR studies with immobilised calmodulin

The SPR studies were performed using a BIAcore 1S + instrument (Cytiva, U.S.A.) with a CM5 sensor chip. S17C-CaM was immobilised in three flow cells of the sensor chip using ligand thiol disulfide exchange coupling. The carboxylated dextran matrix surface was activated by injecting 70 µl of a fresh mixture of 0.1 M NHS and 0.4 M EDC. A reactive disulfide group was introduced by injecting 50 µl of 100 µM PDEA in 0.1 M borate, pH 8.5, followed by 300 µl of 10 µg ml^−1^ S17C-CaM in 10 mM sodium formate, pH 4.3 and finally residual PDEA groups were deactivated by injecting 50 µl of 50 mM l-cysteine, 1 M NaCl, 100 mM sodium formate, pH 4.3. A blank channel for negative control was made by omitting CaM in the coupling step. All four channels were finally blocked by 50 µl of 1 M ethanolamine. The binding and dissociation of wild-type AQP0 and mutants to and from CaM were studied by injecting 200 µl of AQP0 in 20 mM Tris–HCl, 100 mM NaCl, 0.05% DDM, 1 mM CaCl_2_, pH 7.5 at 8 protein concentrations ranging from 110 nM to 3.6 µM over all four flow cells in parallel. The same buffer was used as a running buffer. Dissociations were followed for 120 min and the sensor surface was regenerated by injecting 200 µl 35 mM EDTA, pH 8.0. The flow rate was 20 µl min^−1^ throughout the experiment.

### Analysis of surface plasmon resonance data

The SPR data were subtracted by the contribution from the blank channel and exported as txt files. The data were imported in KaleidaGraph (Synergy Software) for plotting and fitting of user-specified equations. The data could not be satisfactorily fitted without invoking two processes with separate rates, both for the dissociation and association phase. The dissociation phase data were thus fitted using the following equation:

$$R(t)=A_{1}\exp(-k_{\mathrm{off}1}t)+A_{2}\exp(-k_{\mathrm{off}2}t)$$

where *A*_1_ and *A*_2_ are the amplitudes of the two processes and *k*_off1_ and *k*_off2_ are the respective dissociation rate constants. The variable parameters were *A*_1_, *A*_2_, *k*_off1_ and *k*_off2_. The association phase data after blank subtraction were fitted using the following equation:

$$R(t)=C_{1}(1-\exp(-(ck_{\mathrm{on}1}+k_{\mathrm{off}1})t))+C_{2}(1-\exp(-(ck_{\mathrm{on}2}+k_{\mathrm{off}2})t))$$

where *C*_1_ and *C*_2_ are the amplitudes of the two processes s and *k*_on1_ and *k*_on2_ are the respective association rate constants. We thus assume that the fast dissociation process is associated with the fast association process and the slow dissociation process is associated with the slow association process. The variable parameters were *k*_on1_, *k*_on2_, *C*_1_ and *C*_2_.

The two equilibrium dissociation constants, *K_D_*_1_ and *K_D_*_2_, were estimated based on the rate constants as follows:

$$K_{D1}=k_{\mathrm{off}1}/k_{\mathrm{on}1}$$

$$K_{D2}=k_{\mathrm{off}2}/k_{\mathrm{on}2}$$

### Statistical analysis

All experiments were done in triplicates. The significance of the difference between *P_f_*-values and binding constants was estimated using a *Z*-test for population means. The differences were considered significant when *P* < 0.05.

$$Z=\frac{\left(\bar{X_{1}}-\bar{X_{2}}\right)}{\sqrt{\sigma_{1}^{2}+\sigma_{2}^{2}}}$$

## Data Availability

All data are available upon reasonable request from S.T.-H. (susanna.horsefield@biochemistry.lu.se).

## Open Access

Open access for this article was enabled by the participation of Lund University in an all-inclusive *Read & Publish* agreement with Portland Press and the Biochemical Society under a transformative agreement with Bibsam.

## Abbreviations

AQP0: aquaporin-0
CV: column volume
LPR: lipid-to-protein ratio
MST: microscale thermophoresis
SPR: surface plasmon resonance
