# Supplementary material for: The role of phosphorylation in calmodulin-mediated gating of human AQP0
Source: Biochem J. 2024 Jan 4;481(1):17–32. doi: 10.1042/BCJ20230158 (PMC10903448; doi:10.1042/BCJ20230158)
Supplement: Supplementary Material [file BCJ-481-17-s1.pdf]

Table S1. Water permeability, P_f, of wild type AQP0 and AQP0 phospho-mimicking mutants

P_f (µm/s)	Control	wtAQP0	S229D	S231D	S235D
**$CaCl_2$**	142 ± 6.79	365 ± 35.0	343 ± 30.8	315 ± 21.6	345 ± 28.1
**EGTA**	167 ± 7.80	367 ± 25.5	335 ± 13.8	311± 32.6	380 ± 45.6
**$CaCl_2$+CaM**	167 ± 19.3	126± 27.7	306 ± 16.0	331 ± 13.6	352 ± 46.7
**EGTA+CaM**	180 ± 12.9	326 ± 61.0	308 ± 11.4	283 ± 40.5	309 ± 35.0

Supplementary Figures

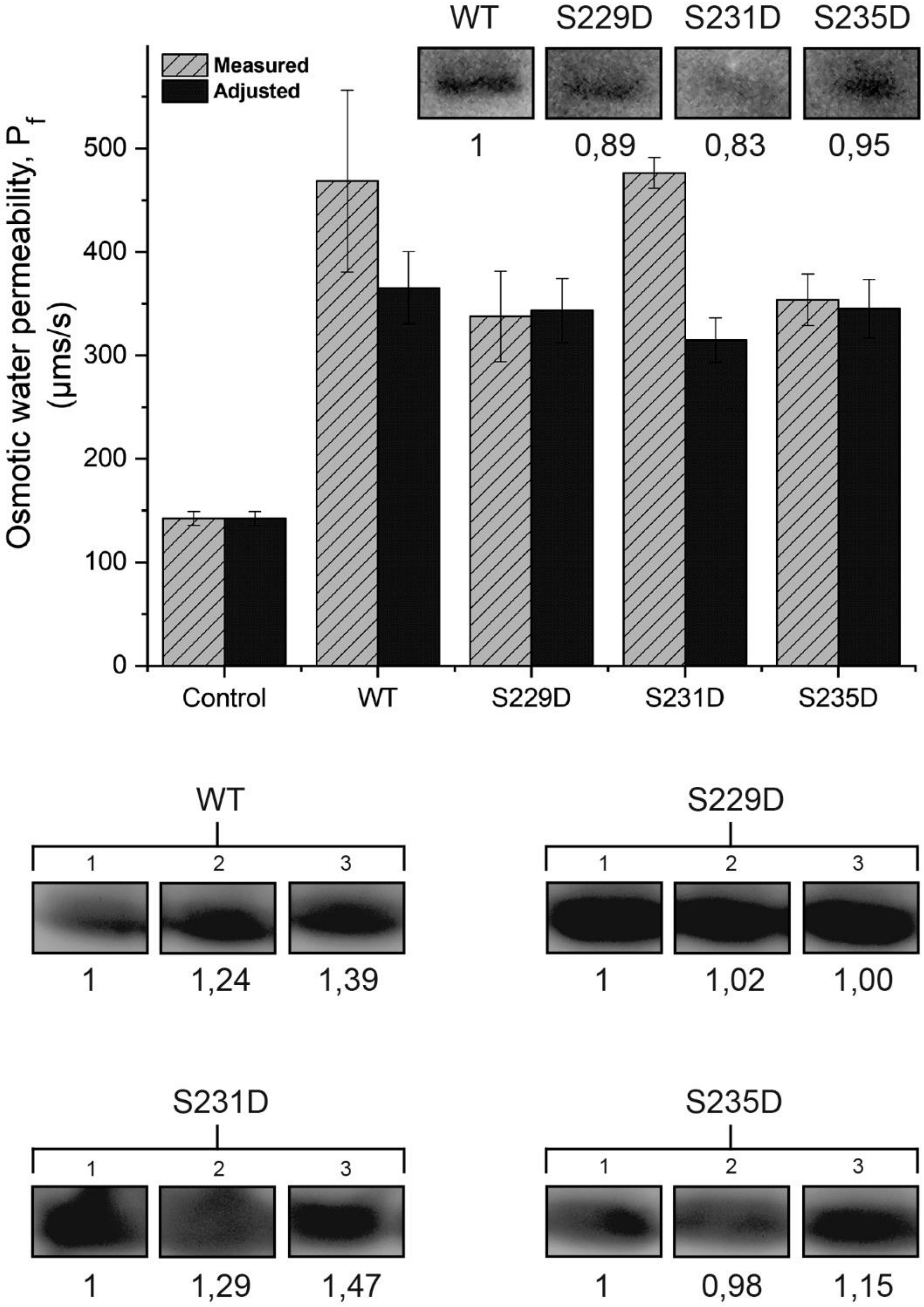

Figure S1. Adjustment of P_f-values for differences in proteoliposome reconstitution efficiency. Top panel: P_f-values for proteoliposomes containing AQP0 constructs and in a buffer containing $CaCl_2$ before (grey bars) and after (black bars) adjustment according to the reconstitution efficiency. The reconstitution efficiency of the AQP0 mutants was determined using Western blot and normalised against wt AQP0 (bar diagram insert). The adjustment correction factor was an average from the three triplicate measurements. **Bottom panel:** The reconstitution efficiency of triplicates of each AQP0 construct was determined using Western blot. All were normalised against the first reconstitution made of each construct. All Western blots were developed using an antibody directed against the His-tag.

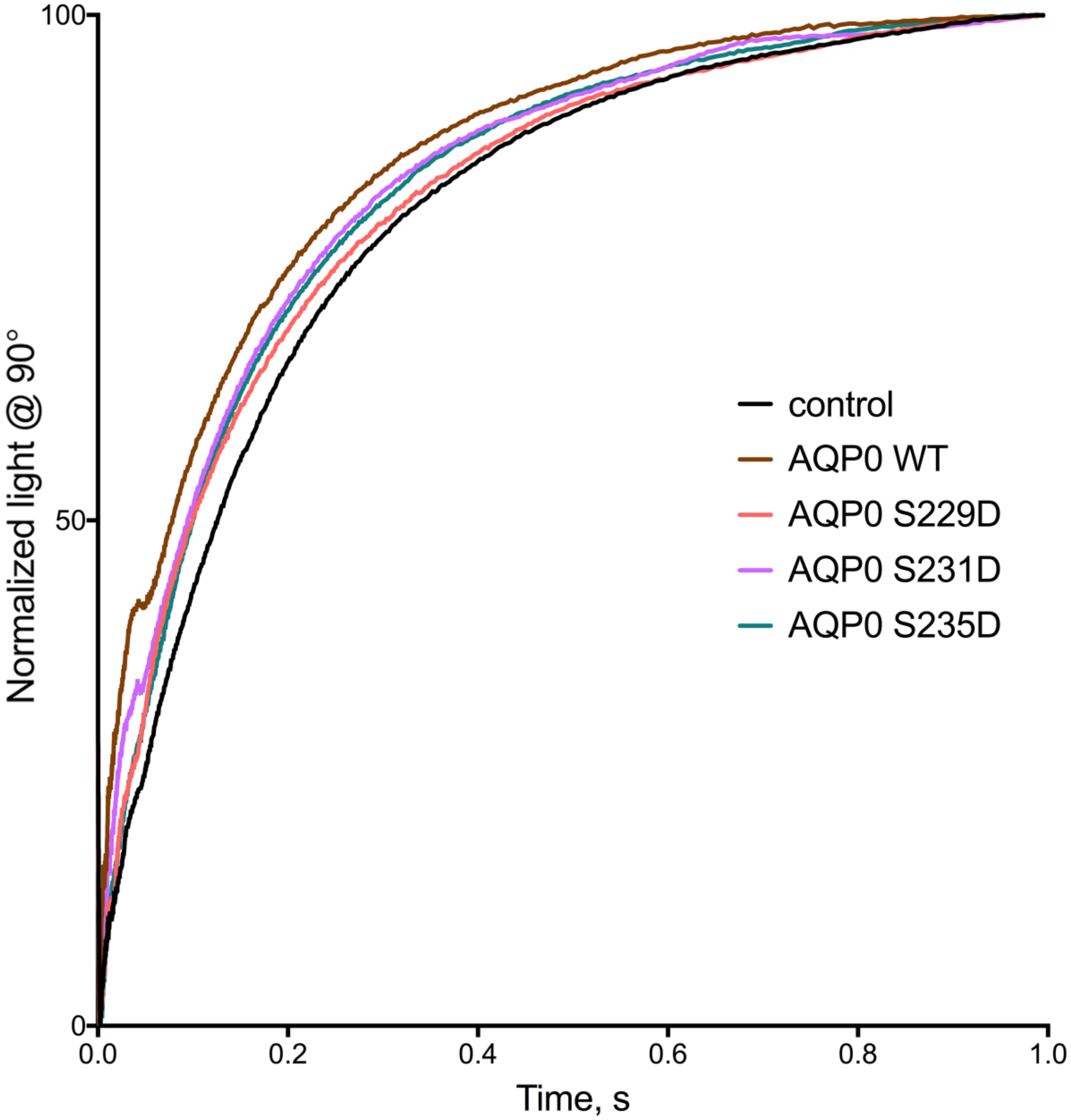

Figure S2. Proteoliposome shrinking assay for AQP0 phospho-mimicking mutants. Typical data and curve fits from the proteoliposome assay for wt AQP0 and phospho-mimicking mutants in the presence of Ca^{2+}. Each curve is the averaged trace from one reconstitution.

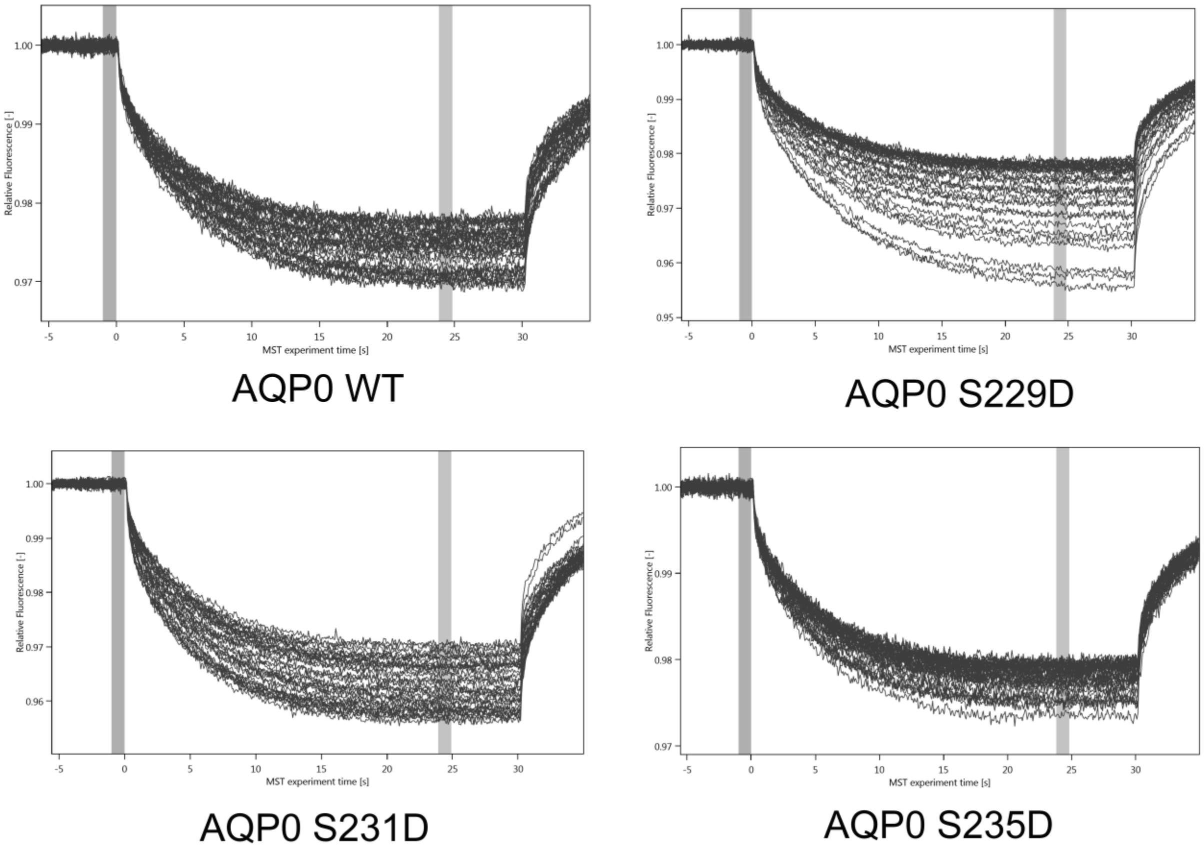

Figure S3. MST data for wt AQP0 and AQP0 phospho-mimicking mutants. Typical MST-traces for wt AQP0 and AQP0 phospho-mimicking mutants. Each trace corresponds to a sample in the AQP0 dilution series. The concentration of labelled CaM was the same in all samples. The difference in relative fluorescence before (right column) and after (left column) heating is used to calculate ΔF_{norm} which can be plotted against the AQP0 concentration to obtain a binding curve.

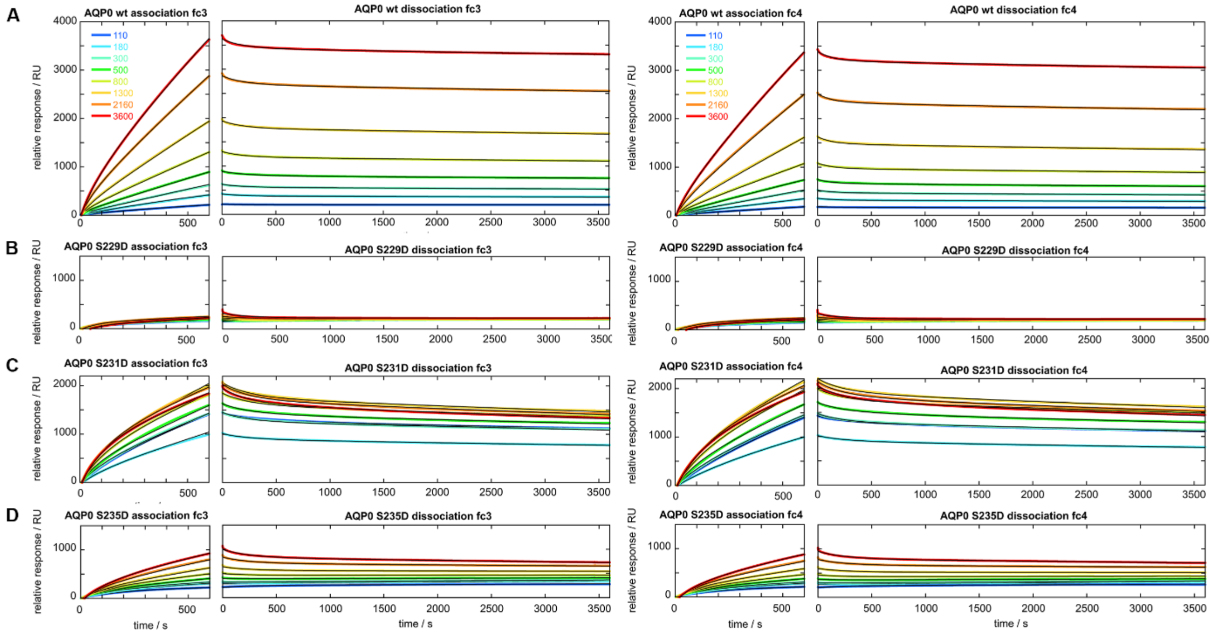

Figure S4. SPR data for AQP0 interaction with calmodulin from flow cells 3 and 4. SPR data for (A) wild-type AQP0, (B) AQP0 S229D, (C) AQP0 S231D and (D) AQP0 S235D). The data are from flow cell 3 and 4 after blank subtraction and shown with equal y-scale with the color codes for the concentration in nM given in the upper left panel. The left panels show association phase data under the injection of AQP0 in constant flow, and the right panels show the dissociation phase data under the following buffer flow. The black lines are fitted bi-exponential curves (see methods).
